# Sacred Cows and Sympathetic Squirrels: The Importance of Biological Diversity to Human Health

**DOI:** 10.1371/journal.pmed.0030231

**Published:** 2006-06-06

**Authors:** Andy Dobson, Isabella Cattadori, Robert D Holt, Richard S Ostfeld, Felicia Keesing, Kristle Krichbaum, Jason R Rohr, Sarah E Perkins, Peter J Hudson

## Abstract

Dobson and colleagues describe how some host species act to reduce the risk of transmission of virulent zoonotic pathogens to humans.

Why are cows sacred? Travel anywhere in India and they have the right of way. Travel anywhere in the eastern United States and you'll see squirrels, more than likely, as roadkill. Yet both species serve a similar epidemiological function: they receive bites from infected vectors that might otherwise have bitten humans, and they break the chain of pathogen transmission. In the case of Indian cattle, the bites are from mosquitoes infected with malaria parasites; the squirrels, on the other hand, receive their bites from ticks infected with the spirochetes that cause Lyme disease. In both cases, the presence of a relatively inefficient host species has reduced the rate of infectious disease spread into the human host population.

Recently, ecologists have uncovered several other ways in which species diversity can benefit human health. In this Essay, we describe how disease risk is influenced by biological diversity and, specifically, how some host species act to reduce the risk of transmission of virulent zoonotic pathogens to people. This represents an exciting area of study where ecologists, conservation planners, and physicians can work together to reduce disease risk and maintain biological diversity. In a world where climate change may allow vector-transmitted diseases to spread from the tropics into the temperate zone, it may be sensible to conserve biological diversity for the purely selfish reasons of protecting human health.

## Zooprophylaxis

One of the oldest examples of biological diversity reducing disease risk occurs with malaria and domestic livestock in India, and it may partly explain why cows are regarded with deep reverence by Hindus. A variety of historical papers have suggested that sleeping in close proximity to domestic livestock, particularly cattle, may reduce the rate at which mosquitoes bite humans, and thus reduce the likelihood of infection with malaria or other vector-borne pathogens (zooprophylaxis; reviewed in [ 1, 2]).

**Figure 1:**
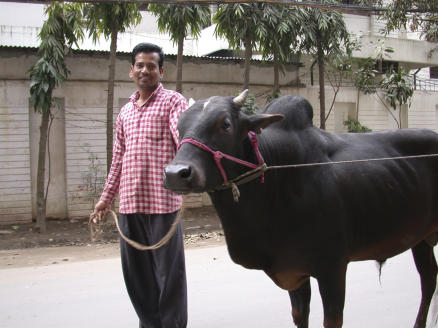
Cow and Proud Owner in Dacca, Bangladesh

We don't specifically know if this is why cows are sacred in parts of India (see Figure 1); they have been considered sacred since the Aryans invaded in the 2nd century, B.C., but many cultural and religious taboos reflect cultural selection for activities that minimize or reduce disease risk. Certainly tribes who spent time in close proximity to cattle might have reduced their risk of malaria, particularly in regions where malaria was transmitted by Anopheles culicifacies (a cattle-biting specialist). Active zooprophylaxis was undertaken when cattle were deliberately used as a barrier between mosquito breeding sites and human settlements; it was probably most widely used in Soviet collective agriculture [ 3] and is again being used in Tanzania today.

A slight problem with this hypothesis is that in many dry areas where malaria exhibits seasonal patterns of abundance, the by-products of cattle supply vital sources of moisture and nutriments that can contribute to the breeding success of mosquitoes. In other words, cattle may divert bites in the short term but increase mosquito abundance in the long term [ 4]. Determining the net effects of species diversity on disease risk in other types of disease systems remains a challenging frontier.

## Loss of Biological Diversity and Disease Risk

A variety of human processes contribute to the loss of biological diversity: habitat loss, habitat fragmentation, overexploitation of populations for food or other economic uses, the introduction of invasive species and diseases, climate change, and pollution [ 5]. Habitat loss is the dominant cause; it is cited as the key cause in around 70% of the species listed as threatened or endangered by the Red List, which records global totals of imperiled species ( http://www.redlist.org). Habitat loss is predominantly driven by the conversion of forests and savannas into agricultural land, cities, and industrial sites. Species are lost by the interaction of two processes: the loss of habitat as conversion proceeds and the fragmentation of the remaining habitat into smaller subdivided patches. Since different species require a minimum area of habitat to meet their energetic and social needs, fragmentation creates many small populations—each of which is highly vulnerable to extinction even when quite large total areas of habitat remain. This creates the central dilemma of conservation biology: species are constantly going extinct locally, but usually only receive major attention when the remaining few individuals are threatened with total annihilation.

Species with larger area requirements tend to be lost first in response to habitat fragmentation (and overexploitation). In the smallest patches, only small species or those with superior dispersal abilities persist. If the predators and competitors that determine the abundance of prey species have disappeared from the smaller patches, then the numbers of prey individuals will increase. If these prey are reservoirs for zoonotic pathogens, then the abundance of these pathogens will also increase. The classic example of this effect occurs with Lyme disease in the forests of the northeastern US—as discussed below, risk of Lyme disease is high in small patches of forest with poor species diversity.

## Squirrels and Lyme disease

**Figure 2:**
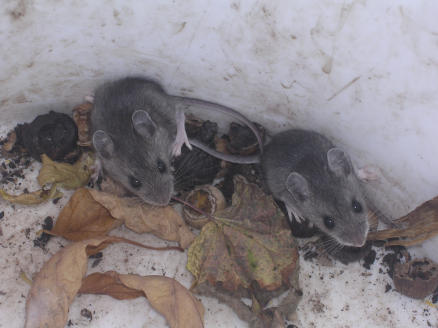
Two Juvenile White-Footed Mice ( Peromyscus leucopus) That Have Been Placed in a Plastic Pail before Being Marked with Ear Tags and Released Mice are important reservoirs for the pathogens that cause Lyme disease, human babesiosis, human granulocytic anaplasmosis, hantavirus pulmonary syndrome, and many other diseases. They thrive in low-diversity vertebrate communities that support few predators and competitors.

Lyme disease is caused by a spirochete bacterium, Borrelia burgdorferi, which is transmitted through the bite of the blacklegged tick ( Ixodes scapularis) in eastern North America. These ticks feed on a wide variety of vertebrate species, including humans. Each of these host species has a different probability of infecting the ticks with the Lyme bacterium. The white-footed mouse ( Peromyscus leucopus) has the dubious distinction of being the most competent reservoir species for the bacterium—over 90% of ticks feeding on wild mice become infected with the Lyme bacterium ( Figure 2). In contrast, fewer than 15% of ticks feeding on gray squirrels ( Sciurus carolinensis) become infected, even though virtually all of the squirrels carry the bacterium [ 6]. As a consequence, the Lyme bacterium is much more prevalent in habitats with many mice than in habitats that harbor a diversity of other species [ 6].

**Figure 3:**
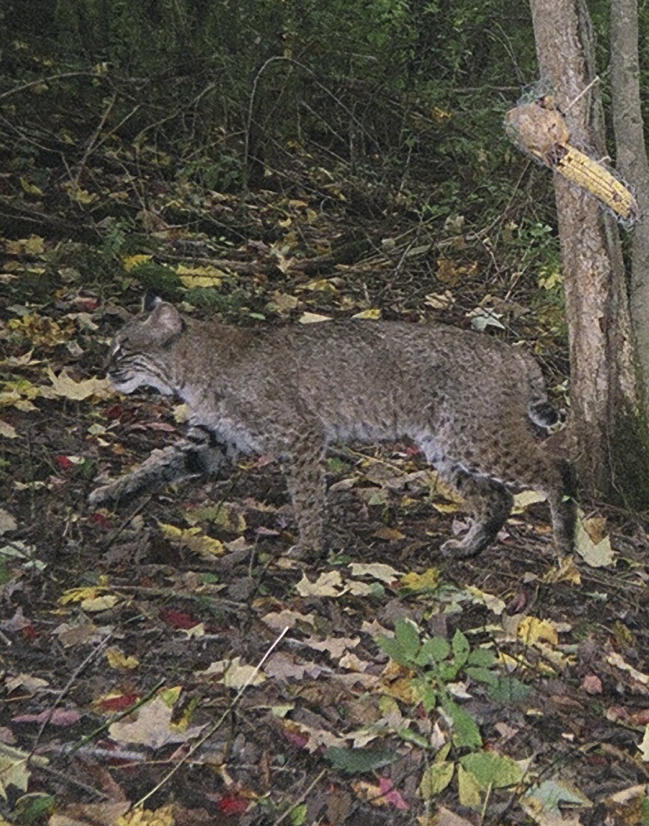
A Bobcat ( Lynx rufus) Drawn to a Scent Lure Placed near an Infrared-Triggered Camera This species is highly sensitive to forest fragmentation, requiring extensive tracts of habitat to support viable populations. Bobcats and other species of mammalian carnivores are important components of diversity that appear to function both as regulators of pathogen reservoirs such as white-footed mice and as hosts that might deflect tick meals away from more competent reservoirs for zoonotic pathogens.

Where are white-footed mice most common? Several studies have shown that very small patches of forest (less than about two hectares) contain high densities of white-footed mice. These patches are too small to support the predators and competitors that typically determine mouse numbers ( Figure 3). So in small patches of forest, ticks have almost nothing to feed on except white-footed mice, and there is an overabundance of these. Small fragments of forest boast some of the highest Lyme disease risk ever documented [ 7]. In contrast, bigger patches of forest harbor squirrels, chipmunks, foxes, weasels, and coyotes—these are all poor reservoir species for Lyme disease and also reduce white-footed mouse abundance. Here, high species diversity appears to both regulate the most competent Lyme disease reservoir (mice) and deflect tick meals away from mice and toward less competent reservoirs.

## Yellow-Necked Mice and Tick-Borne Encephalitis

Tick-borne encephalitis (TBE) is a viral infection that circulates among free-living yellow-necked mice ( Apodemus flavicollis) of Europe and the former Soviet Union. When an infectious tick bites a human, the pathogen spills over, causing serious illness. The virus attacks the human central nervous system, causing meningitis and encephalitis. Woodland workers are often at risk, but some of the “hot spots” for TBE transmission are also key holiday locations where children have become infected and died.

The intriguing part of the transmission process is that the wild zoonotic hosts—the yellow-necked mice—do not become viremic, but permit transmission of the virus between co-feeding ticks through a nonviremic process. Successful transmission requires that an infectious nymph bites a mouse at the same time as a susceptible larva is feeding [ 8]. When the density of mice is high, then the probability of two ticks feeding on the same host at the same time is very small, so transmission declines to levels where the pathogen cannot persist. In contrast, when the mice are at very low density, not enough infectious ticks are produced for the disease to persist. So the density of mice acts as a major determinant of transmission; this only allows TBE to persist when mouse density is moderate.

Concomitantly, a second determinant of transmission success is mediated by the presence of noncompetent hosts in the system. Adult ticks don't bite mice, so the presence of large mammals (usually deer) is necessary to sustain the tick population, but deer do not permit successful transmission of TBE [ 9]. Once again, the relative density of these hosts is important in determining the level of disease risk to humans. When deer density is high, disease levels are low because a high proportion of ticks are feeding on deer, and virus is lost from the system. When we combine these two dimensions of dilution, then high biodiversity and high densities of hosts are good for human health.

## House Sparrows and West Nile Virus

Similar ecological forces seem to operate with West Nile virus (WNV). This disease first appeared in the US in New York in 1999 when significant numbers of birds, particularly crows, began dropping dead in and around New York City. Quite soon afterward, the first human cases were reported, and several of these patients died [ 10]. In the next five years, WNV spread to cover almost the entire US and parts of Canada, Mexico, and Central America.

The virus is transmitted by a diversity of mosquito species and can replicate in a variety of bird species. Although in some host species the pathology is undetectable, in others, particularly crows, a rapid viremia leads to death in only a couple of days. Humans, horses, and alligators are probably dead-end hosts, meaning that viremia is either too modest or too transient to provide a source of infection for later-feeding mosquitoes.

Teasing out whether species diversity affects WNV dynamics will be a thorny problem, as a large number of host and vector species are involved. Several authors have presented initial analyses that suggest there is a decline in prevalence of human WNV cases in areas with high avian diversity [ 11]. WNV seems to pose an analogous situation to Lyme disease in that the most competent reservoirs—house sparrows, house finches, American robins, blue jays, and grackles—proliferate in heavily fragmented or otherwise degraded habitats. Consequently, where bird diversity is low, the bird community consists largely of competent reservoirs, but where natural habitats are largely intact and bird diversity is higher, many incompetent reservoirs dilute and disrupt the transmission cycle of the virus.

Although a protective role for high biological diversity has been supported for directly transmitted pathogens such as hantaviruses, we expect that species diversity is most likely to reduce disease risk with vector-transmitted infections. The primary reason we get disease reductions is that the vectors that transmit the pathogen only take a limited number of bites in their lifetime; when some of these bites are taken from hosts that are not competent to amplify the pathogen, these bites are wasted. This reduces the rate at which the pathogen is transmitted. Ecologists have termed this phenomenon, the “dilution effect” [ 12–14]. The primary process that produces a dilution effect is the increased diversity of host species that increase the proportion of bites that are wasted.

From one crucial perspective, these transmission dilution effects may be even stronger for WNV than for Lyme disease or TBE. In the case of ticks that transmit Lyme disease and TBE, the prolonged blood meal that the tick receives when it feeds as an adult on large mammalian hosts acts as its primary form of nutrition for tick egg production. For this reason, tick abundance may be determined at least in part by the abundance of the large host species, but, at the same time, these are the species that tend to be noncompetent and unable to either amplify or transmit the pathogen. Therefore, some components of diversity, such as deer, might simultaneously reduce infection prevalence of ticks and boost tick numbers—leading to mixed effects on disease risk. In the case of tick-borne diseases, therefore, species richness alone may be only part of the story, and we need to encompass both species diversity and abundance to identify how wild animal populations influence disease risk to humans.

In contrast, the abundance of mosquitoes is often independent of the abundance of the hosts that provide female mosquitoes with blood meals. Although these blood meals are crucial to egg production, the local abundance of mosquitoes may be more strongly dependent on the abundance of pools of water in which to lay eggs. For mosquito-borne zoonoses, therefore, host diversity, per se, is more likely to influence disease risk.

## Palliative Plants

Agricultural scientists have been interested in the effects of plant diversity on agricultural diseases for many decades, and several studies have shown that crop diversity can reduce the total burden of disease in agricultural systems. For example, the transmission of plant pathogens that specialize on particular crops can be reduced simply by interspersing other crop species that the pathogen does not readily infect. In essence, the nonhost plants can act as physical barriers to the dispersal of the pathogen, absorbing them without expressing disease or transmitting them further [ 15].

A classic example of the potential effects of crop diversity on disease was found in corn. In experimental fields, the presence of a diversity of noncorn plants reduced incidence of corn smut, a fungal pathogen [ 16]. More recently, diverse mixtures of rice varieties showed both a lower incidence of rice blast disease and a greater yield than did monocultures of a single rice variety [ 17]. The role of high plant diversity in reducing agricultural disease transmission provides strong analogies to zoonotic systems. Plant species that are incompetent pathogen reservoirs can reduce transmission by regulating the size of the host population (after all, space taken up by incompetent reservoirs can't be colonized by the competent ones). Diverse species in both types of systems can absorb but do not transmit infections.

## Predators and Chronic Wasting Disease

These plant studies illustrate the potential role that adding competitors can have on disease risk, but reductions in disease prevalence also occur in situations when predators are added to an ecological community. For example, we have long known from studies of predators feeding on Dall Sheep or Serengeti wildebeest that predators selectively prey upon sick and diseased individuals that are easier to capture [ 18]. The removal of these sick animals before they die from the infection removes the most heavily infected individuals from the host population and reduces parasite transmission rates. The classic example of this occurs with red grouse, gamebirds in northern Britain. Here, the presence of parasitic nematodes increases the vulnerability of birds to foxes and to birds of prey. When the predators remove these heavily infected birds, then the infective stages the parasites would have produced are removed from the system and the net parasite abundance in the surviving bird population is reduced, leading to a rise in the bird population. The predators are actually making the grouse population healthier and are leading to an increase in their abundance, as long as the predator is a specialist [ 19].

Similar effects may occur with the recently emerging spongiform encephalopathies of deer (chronic wasting disease) and domestic livestock (bovine spongiform encephalopathy, or “Mad Cow Disease”), which are threatening the future of the agricultural industries worldwide. Either of these could potentially cross the species barrier to humans, causing variant Creutzfeldt-Jacob Disease or chronic brain wasting disease. The primary mode of transmission for these pathogens appears to occur when infected livestock die (usually over winter). Transmission from the infected carcasses occurs when the carcasses are gnawed upon by other creatures that are nutritionally stressed.

A curious feature of the spongiform encephalopathies is that their natural range is usually in an area with very poor soil, where hosts naturally suffer bone mineral deficiencies. When scavengers, such as coyotes and buzzards, are abundant, the carcasses will not last long, and it's unlikely that they will be available for transmission. However, when coyote and buzzard numbers are reduced by game managers, the carcasses can persist in the environment, and transmission rates might allow the pathogens to both increase in prevalence and establish themselves in new regions. Intriguingly, dogs seem to be totally resistant to prions. Although many dogs in the United Kingdom probably ingested infected beef, there are no veterinary reports of spongiform encephalopathies in dogs; several records occur for cats. This absence of dog cases may reflect past selection for deletion of prion susceptibility in canid species that obtain significant amounts of their food from scavenging or preying upon weakened individuals.

## Biological Diversity and Global Climate Change

Studies of the effects of diversity on disease are providing important insights into the major role that ecological communities play in regulating the natural abundance of zoonotic pathogens that infect humans and their domestic livestock. The problems are scientifically challenging because they involve understanding the dynamics of complex multispecies systems where birth and death rates operate on a variety of different time scales. Given that significant threats to human health may be buffered by the presence of a diversity of other species, we need to understand the dynamics of species interactions. Unfortunately, this need is increasingly urgent because we are losing biological diversity at the fastest rate ever recorded.

Understanding species interactions is particularly important (or urgent) when we consider how the world of infectious diseases is likely to change in the face of ongoing climate change. At present, vector-borne diseases of humans are much more prevalent in the tropics. Tropical infections such as malaria, sleeping sickness, dengue fever, Chagas disease, leishmaniasis, and yellow fever are all diseases that worry Western tourists and military planners. The main health and economic impact of these diseases is felt by the people who live in the world's poorest tropical countries, and many would argue that these pathogens are the principal economic constraint on these countries [ 20]. The warm and humid climates of the tropics provide ideal conditions for many vectors and pathogens. As the world becomes warmer, many of these pathogens may be able to spread beyond the tropics [ 21].

Here we suddenly discover a supreme irony: although vector-transmitted diseases take a significant toll on human health in the tropics, this toll may be significantly buffered by the presence of the large diversity of other species with which tropical people coexist. Now, as we convert habitats for agriculture or with urbanization, we improve human access to food and infrastructure, but we may also reduce the ability of natural systems to buffer disease. How much worse will things get in the tropics as biodiversity declines there?

Finally, we should note that as vector-transmitted diseases disperse into the current temperate zones, they will not only benefit from a wetter and warmer world, but also from one in which the natural level of biodiversity is lower than in the tropics. Certainly, some host species may also spread from the tropics into the temperate zone, but larger species typically spread at a slower rate than smaller ones, so for a significant time, vector-transmitted diseases will be moving down a gradient of biodiversity. Given a restricted choice of hosts on which to feed, they are likely to focus their attention on the most common and most abundant species: humans, and their domestic animals and plants. This provides a strong selfish motivation to conserve biological diversity—our health may depend upon it.

## Abbreviations

TBE: tick-borne encephalitis
WNV: West Nile virus

## References

[pmed-0030231-b1] Service MW (1991). Agricultural development and arthropod-borne diseases: A review. Rev Saude Publica.

[pmed-0030231-b2] Sota T, Mogi M (1989). Effectiveness of zooprophylaxis in malaria control: A theoretical inquiry, with a model for mosquito populations with two bloodmeal hosts. Med Vet Entomol.

[pmed-0030231-b3] World Health Organization (1991). Joint WHO/FAO/UNEP panel of experts on environmental management for vector control.

[pmed-0030231-b4] Bouma MJ, Rowland M (1995). Failure of passive zooprophylaxis: Cattle ownership in Pakistan associated with a higher prevalence of malaria. Trans R Soc Trop Med Hyg.

[pmed-0030231-b5] Soule ME (1991). Conservation: Tactics for a constant crisis. Science.

[pmed-0030231-b6] LoGiudice K, Ostfeld RS, Schmidt KA, Keesing F (2003). The ecology of infectious disease: Effects of host diversity and community composition on Lyme disease risk. Proc Natl Acad Sci U S A.

[pmed-0030231-b7] Allan BF, Keesing F, Ostfeld RS (2003). Effects of habitat fragmentation on Lyme disease risk. Conserv Biol.

[pmed-0030231-b8] Randolph SE, Miklisová D, Lysy J, Rogers DJ, Labuda M (1999). Incidence from coincidence: Patterns of tick infestations on rodents facilitate transmission of tick-borne encephalitis virus. Parasitology.

[pmed-0030231-b9] Rosa R, Pugliese A, Norman RA, Hudson PJ (2003). Thresholds for disease persistence in models for tick-borne infections including non-viraemic transmission, extended feeding and tick aggregation. J Theor Biol.

[pmed-0030231-b10] Campbell GL, Marfin AA, Lanciotti RS, Gubler DJ (2002). West Nile virus. Lancet Infect Dis.

[pmed-0030231-b11] Ezenwa VO, Godsey MS, King RJ, Gupthill SC (2006). Avian diversity and West Nile virus: Testing associations between biodiversity and infectious disease risk. Proc R Soc Lond B Biol Sci.

[pmed-0030231-b12] Ostfeld RS, Keesing F (2000). Biodiversity and disease risk: The case of Lyme disease. Conserv Biol.

[pmed-0030231-b13] Norman R, Bowers RG, Begon M, Hudson PJ (1999). Persistence of tick-borne virus in the presence of multiple host species: Tick reservoirs and parasite-mediated competition. J Theor Biol.

[pmed-0030231-b14] Keesing F, Holt RD, Ostfeld RS (2006). Effects of species diversity on disease risk. Ecol Lett.

[pmed-0030231-b15] Burdon JJ, Chilvers GA (1982). Host density as a factor in plant disease ecology. Annu Rev Phytopathol.

[pmed-0030231-b16] Pitre HN, Boyd FJ (1970). A study of the role of weeds in corn fields in the epidemiology of corn stunt disease. J Econ Entomol.

[pmed-0030231-b17] Zhu Y, Hairu Chen, Jinghua Fan, Yunyue Wang, Yan Li (2000). Genetic diversity and disease control in rice. Nature.

[pmed-0030231-b18] Packer MJ, Holt RD, Hudson PJ, Lafferty KD, Dobson AP (2003). Keeping the herds healthy and alert: Implications of predator control for infectious disease. Ecol Lett.

[pmed-0030231-b19] Hudson PJ, Dobson AP, Newborn D (1992). Do parasites make prey vulnerable to predation? Red grouse and parasites. J Anim Ecol.

[pmed-0030231-b20] Sachs J (2005). The end of poverty.

[pmed-0030231-b21] Harvell CD, Mitchell CE, Ward JR, Alitzer S, Dobson AP (2002). Climate warming and disease risks for terrestrial and marine biota. Science.

